# The SARS-CoV-2 viral load in COVID-19 patients is lower on face mask filters than on nasopharyngeal swabs

**DOI:** 10.1038/s41598-021-92665-3

**Published:** 2021-06-29

**Authors:** Agnieszka Smolinska, David S. Jessop, Kirk L. Pappan, Alexandra De Saedeleer, Amerjit Kang, Alexandra L. Martin, Max Allsworth, Charlotte Tyson, Martine P. Bos, Matt Clancy, Mike Morel, Tony Cooke, Tom Dymond, Claire Harris, Jacqui Galloway, Paul Bresser, Nynke Dijkstra, Viresh Jagesar, Paul H. M. Savelkoul, Erik V. H. Beuken, Wesley H. V. Nix, Renaud Louis, Muriel Delvaux, Doriane Calmes, Benoit Ernst, Simona Pollini, Anna Peired, Julien Guiot, Sara Tomassetti, Andries E. Budding, Frank McCaughan, Stefan J. Marciniak, Marc P. van der Schee

**Affiliations:** 1 Owlstone Medical Ltd., Cambridge, Cambridgeshire UK; 2 Department of Pharmacology and Toxicology, Maastricht University, Maastricht, The Netherlands; 3 inBiome B.V., Amsterdam, The Netherlands; 4 Cambridge Clinical Laboratories Ltd., Cambridge, Cambridgeshire UK; 5 Cambridge University Hospitals NHS Foundation Trust, Addenbrooke’s Hospital, Cambridge, UK; 6 Department of Medicine, Addenbrooke’s Hospital, Cambridge, UK; 7 University of Cambridge, Cambridge, UK; 8 Pulmonology, OLVG, Amsterdam, The Netherlands; 9 Department of Medical Microbiology, Maastricht University Medical Center, Care and Public Health Research Institute (Caphri), Maastricht, The Netherlands; 10 Repiratory Department, CHU Liège, Liège, Belgium; 11 Department of Experimental and Clinical Medicine, University of Florence, Florence, Italy; 12 Microbiology and Virology Unit, Careggi University Hospital, Florence, Italy; 13 Department of Experimental and Clinical Biomedical Sciences “Mario Serio”, University of Florence, Florence, Italy; 14 Interventional Pulmonology Unit, Careggi University Hospital, Florence, Italy

**Keywords:** Infectious-disease diagnostics, Diagnostic markers, Infectious diseases

## Abstract

Face masks and personal respirators are used to curb the transmission of SARS-CoV-2 in respiratory droplets; filters embedded in some personal protective equipment could be used as a non-invasive sample source for applications, including at-home testing, but information is needed about whether filters are suited to capture viral particles for SARS-CoV-2 detection. In this study, we generated inactivated virus-laden aerosols of 0.3–2 microns in diameter (0.9 µm mean diameter by mass) and dispersed the aerosolized viral particles onto electrostatic face mask filters. The limit of detection for inactivated coronaviruses SARS-CoV-2 and HCoV-NL63 extracted from filters was between 10 to 100 copies/filter for both viruses. Testing for SARS-CoV-2, using face mask filters and nasopharyngeal swabs collected from hospitalized COVID-19-patients, showed that filter samples offered reduced sensitivity (8.5% compared to nasopharyngeal swabs). The low concordance of SARS-CoV-2 detection between filters and nasopharyngeal swabs indicated that number of viral particles collected on the face mask filter was below the limit of detection for all patients but those with the highest viral loads. This indicated face masks are unsuitable to replace diagnostic nasopharyngeal swabs in COVID-19 diagnosis. The ability to detect nucleic acids on face mask filters may, however, find other uses worth future investigation.

## Introduction

The year 2020 ended with nearly 80 million confirmed cases and more than 1.75 million deaths worldwide due to the coronavirus disease 2019 (COVID-19). More than 10 vaccines have been approved by one or more jurisdictions globally and are being administered to curb the spread of COVID-19^1^. Since the beginning of the pandemic, numerous diagnostic tests have received emergency use authorization from the US Food and Drug Administration^2^. Many of the tests depend on nucleic acid amplification workflows for detection of specific gene regions of SARS-CoV-2, the causative agent of COVID-19, but other testing modalities, including serological assays for reactive antibodies and viral antigens, and technologies such as computed tomography (CT) scans, biosensors, genetic sequencing, immunoassays, smartphone sensors, and others have been developed^2–5^.

Breathing, talking, singing, and coughing release respiratory droplets with a range of sizes and masses^6,7^. Exhaled droplets can transmit SARS-CoV-2 and, with sufficiently high viral loads and exposures, can lead to new COVID-19 infections. Larger coarse aerosols have limited airborne range before they fall to the ground^8^. Smaller fine breath aerosols can transport respiratory viruses through the air over long distances and, since airborne SARS-CoV-2 has a half-life of hours, appear to contribute to COVID-19 infections in some circumstances^9–12^.

Three main routes for respiratory virus transmission are direct contact with an infected person or contaminated surface, encounters with coarse aerosols surrounding an infected individual, and contact with fine aerosols^12^, which become more dilute with increasing distance from the source in well-ventilated conditions^13^. For most cases of COVID-19 the mode of viral transmission is not known and so debate continues about the relative contribution of the three major routes to disease spread. COVID-19 infection via airborne transmission of SARS-CoV-2 depends on variables such as aerosol size, viral load, half-life of aerosolized virus, and level of aerosol-generating activity^14^.

Nasopharyngeal swabs (NPS) are the dominant sample matrix for nucleic acid amplification tests for COVID-19 diagnosis^2^. Another area of interest is the potential to use air filtration materials from personal protective equipment (PPE), such as face masks or respirators, for diagnostic testing as demonstrated for rhinovirus and influenza detection^15,16^. Recent work has shown that the coronavirus load emitted by symptomatic individuals into the environment is lower when wearing than not wearing a mask^17,18^. The research by Leung et al*.* showed that face masks were very effective at reducing the already low level of viral shedding in both coarse (> 5 µm diameter) and fine (≤ 5 µm diameter) exhaled aerosols of symptomatic individuals^17^. The results suggest that surgical masks could prevent transmission of human coronaviruses and influenza viruses and imply that face masks retain viral particles and, therefore, may be suitable for virus detection.

To understand whether filters embedded in PPE can be used to detect SARS-CoV-2, a trial to test the hypothesis that face mask filters are non-inferior to NPS for quantitative polymerase chain reaction (qPCR)-based detection of SARS-CoV-2 was conducted. For that purpose, a nebulizer test system was developed for the controlled generation of small aerosol particles and used to disperse inactivated coronaviruses (SARS-CoV-2 and HCoV-NL63) onto electrostatic filters to measure the limit of detection. Following laboratory-based testing of filters for SARS-CoV-2 detection, hospitalized patients with confirmed COVID-19 were recruited to give samples for comparative diagnostic testing of non-invasive samples collected by breathing into a face mask with NPS by a nucleic acid amplification workflow.

## Materials and methods

### COVID-19 patient recruitment, ethics, and confidentiality

This study (ClinicalTrials.gov Identifiers: NCT04467112, NCT04508556) was initiated by Owlstone Medical Ltd. as a non-inferiority trial to compare the diagnostic accuracy of samples collected on face mask filters with standard of care NPS samples tested by quantitative reverse transcription-polymerase chain reaction (qRT-PCR). The identity of sample donors was known only to the clinical staff involved in patient care and sample collection. Samples were assigned anonymous sample identifiers and no patient-identifying information was shared with Owlstone Medical or external testing affiliates.

Patients were recruited from university hospitals and each site received written ethics approval for protocols complying to the Declaration of Helsinki and local regulations for clinical research; the approval details are as follows: United Kingdom (NHS HRA London—Camberwell St Giles Research Ethics Committee REC reference: 20/LO/0581), Netherlands (Advisory Committee for Scientific Research approval number: ACWO 20u.420/JW/WO 20.139), Belgium (Comité d’Ethique Hospitalo-Facultaire Universitaire de Liège Nr belge: B7072020000007), and Italy (Comitato Etico Regionale per la Sperimentazione Clinica della Regione Toscana approval number: 18099_bio). In-hospital patients with confirmed COVID-19 were asked to participate in this study and provided informed consent. Patients were eligible to participate if they were older than 18 (> 16 years in the UK), had a positive COVID-19 diagnosis by NPS qRT-PCR, and were able to provide face mask filter and follow-up NPS samples. Patients requiring oxygen via a mask, needing > 4 L/min oxygen via nasal cannula, deemed at risk of Type II respiratory failure, receiving inotropic medications, or who could not provide consent or could not understand and comply with the instructions for breath sample collection were excluded.

### Construction and validation of an aerosol generating system

**Figure 1:**
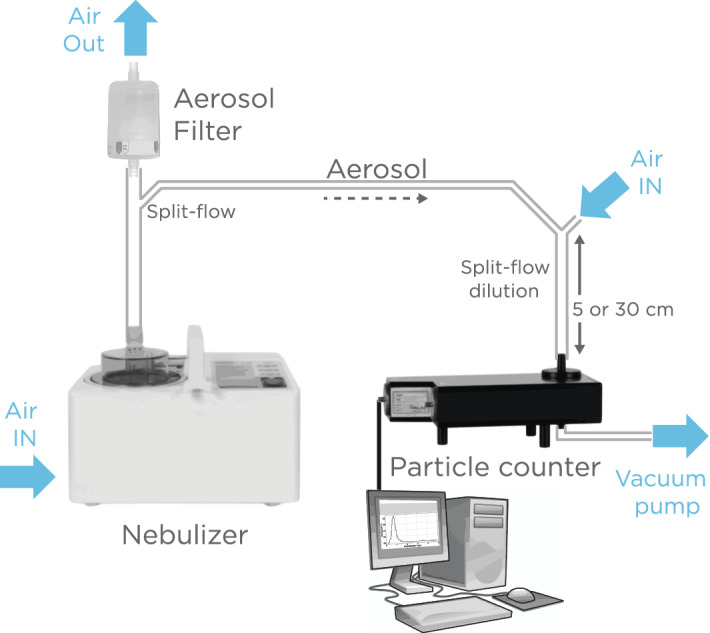
An aerosol generating system connected to a particle counter was used to characterize aerosols. Particles generated by aerosolizing a test solution using an OMRON nebulizer were mixed with air and sized with a Welas particle counter.

An OMROM NE-U780 ultrasonic nebulizer was used to produce a steady stream of aerosolized fluid from a reservoir (Fig. 1). The generated aerosol concentration was around 8 × 10^4^ particles/cm^3^ which was then diluted to 2.2 × 10^4^ particles/cm^3^ using two split-flow dilution sections. A Welas 2100 HP optical particle counter was used to characterize the aerosol. To avoid contamination of the particle counter, only deionized water and buffer solution, without virus, were aerosolized and measured. Since the aerosolization rate and droplet size is dependent on viscosity, surface tension, and density, the generated aerosol showed little variation with the introduction of a trace amount of virus. The particle counter drew, via a vacuum pump, a constant 5 L/min of diluted aerosol/air mixture through a detection chamber and the particles were sized by optical scattering. The measured particle size counts were placed into 59 logarithmically spaced bins between 0.143 and 10.0 µm. The aerosol particle distributions were measured at two different distances, 5 cm and 30 cm, between the dilution region and the particle counter to measure the evaporation of the droplets when mixed with ambient air. The 5 cm path length corresponded to shortest distance for which it was feasible to connect the split flow to the particle counter whereas 30 cm represented fixed distance between dilution region and the filter based on the constraints of the filter holder.

The aerosol, as produced by the nebulizer and measured 5 cm from the dilution region, had a concentration of 7.5 ng/cm^3^ and a mean particle size by mass of 2.4 µm. The same aerosol when measured 30 cm from the dilution region had a concentration of 2.4 ng/cm^3^ and a mean particle size by mass of 0.86 µm. This corresponds to a threefold decrease in measured aerosol mass, which agrees with work done by Morawska et al*.*^19^.

### Dispersion of coronaviruses onto filters

**Figure 2:**
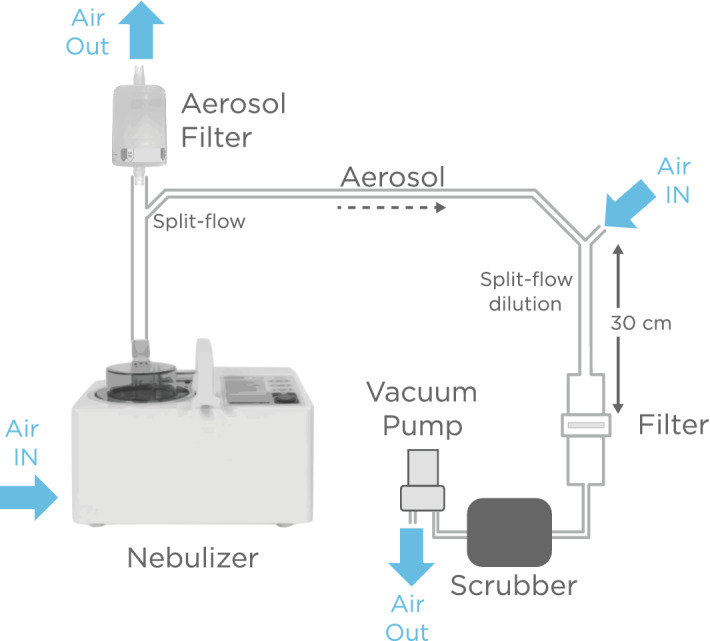
The aerosol generating system was adapted to disperse inactivated virus onto filters. Particles containing inactivated virus diluted in DMEM were generated using an OMRON nebulizer, mixed with air and drawn onto an electrostatic filter by a vacuum pump.

Dispersion of aerosolized virus on filters was accomplished by replacing the particle counter by an assembly that housed an electrostatic filter (Fig. 2, Supplementary Fig. 1). The distance from the filter to the split-flow dilution region was 30 cm, and hence the droplets underwent evaporation to a smaller size. 5 L/min of diluted aerosol/air mix was drawn through the electrostatic filter via a vacuum pump to achieve the same flow conditions as measured by the particle counter. All exhausts were filtered to prevent viruses from reentering the system from the ambient intake. Inactivated SARS-CoV-2 and HCoV-NL63 viruses were diluted with DMEM propagation medium to final known concentrations.

### Limit of detection and quantification of aerosolized SARS-CoV-2 and HCoV-NL63

SARS-CoV-2 is the coronavirus behind COVID-19 whereas human coronavirus HCoV-NL63 is a coronavirus that causes common-cold symptoms in vulnerable populations^20^. SARS-CoV-2 and HCoV-NL63 were propagated and inactivated (Supplementary Method 1). The inactivated viruses were diluted in DMEM medium to 5000, 500, 50, and 5 copies/µL, and 5 min of aerosolization was calculated to lead to the expected delivery or 1000, 100, 10, and 1 copy/filter, respectively. Testing of each dilution was performed in six replicates and included a DMEM-only blank between each dilution. Viral RNA was extracted from filters by adding 4 mL of lysis buffer (Chemagic Viral Lysis Buffer (CMG-832); PerkinElmer Chemagen Technologie GmbH) and vortexing the sample for 2 min. Three-hundred-fifty microliters of the sample extract were used for RNA extraction (FavorPrep Tissue Total RNA Isolation Kit; Favorgen) and eluted in 80 µL. Subsequently 5 µL were used for qRT-PCR (QuantStudio5).

#### RNA standard curves

Isolated RNA (Covid-19 and HCoV-NL63; PAMM) was used to make (tenfold) serial dilutions in RNase-free water (from 10^8^ to 10 copies/µL). 5 ul of each dilution was used for qRT-PCR in order to monitor reverse transcriptase efficiency.

#### DNA standard curves

Plasmid DNA (pEX-A128-nCoV_all; 3033 bp; Eurofins and p1055; pGEM-Teasy containing HCoV-NL63 sequences) was diluted to 10^10^ copies/µL. This stock solution was used to make (tenfold) serial dilutions and served as a PCR control.

qRT-PCR cycle threshold (Ct) calibration curves for SARS-CoV-2 and HCoV-NL63 viral RNA were generated using 100 µL of inactivated virus sample in DMEM medium for RNA isolation followed by elution in 100 µL RNase-free H_2_O. These RNA isolates were serially diluted to obtain concentrations of 10 to 10^9^ copies/µL to make standard Ct curves for qRT-PCR of SARS-CoV-2 and HCoV-NL63.

### Face mask filter and nasopharyngeal swab collection

Samples were collected from 48 individuals who provided consent to participate in the project. PPE-wearing clinical personnel supervised patients in fitting the face mask, holder, and head strap assembly. Patients placed the face mask over nose and mouth and then secured the fit by adjusting the fasteners on the head straps attached to the plastic face mask holder. Patients were instructed to breath normally into the face mask for between 30 and 60 min during which they were asked to cough 10 times and speak out loud for 1 min. The duration of the breath sampling session was entered into the electronic case report form.

Following collection of the exhaled breath sample, the head straps were loosened, and the face mask removed. The electrostatic filter (Hollingsworth Technostat T150/15), removed from the face mask using forceps, was deposited in a leakproof polypropylene container containing 4 mL of DNA/RNA Shield (Zymo Research, Irvine, CA, USA). Shortages of leakproof containers, due to worldwide COVID-19 demand, led to the need for filters from one site to be stored at − 80 °C and shipped frozen, without DNA/RNA Shield, following collection. Testing of face mask filters extracted immediately or extracted 5 days after being stored in the dry, frozen state did not reveal any statistically significant differences in qRT-PCR Ct values (Supplementary Fig. 2).

After breath sample collection, clinical personnel collected NPS samples which were placed in a polypropylene vial containing 1 mL of DNA/RNA Shield after collection. Follow-up NPS samples were not collected for 8 patients, leading to incomplete results (Supplementary Data). Filters and NPS samples in DNA/RNA Shield were shipped at ambient temperature to a reference laboratory for SARS-CoV-2 testing. Samples stored in DNA/RNA Shield were shipped in 6 separate batches from October 2020 to December 2020. The ambient temperature range over those 6 dates and locations spanned a low of 27° F (− 3 °C) and high of 57° F (14 °C). Filters collected without DNA/RNA Shield were frozen after collection and were shipped to the testing laboratory on dry ice.

### Face mask filter and nasopharyngeal swab testing for SARS-CoV-2

For RNA isolation, a 300 µL aliquot of the DNA/RNA Shield solution was taken from NPS and filter samples, except for filters that were stored and shipped in the dry, frozen state. The dry, frozen filters were soaked in 4 mL of Chemagen lysis buffer before aliquoting 300 µL for RNA isolation. RNA was isolated using a Chemagic-360 instrument (PerkinElmer) and Chemagic Viral DNA/RNA kits (cat. no CMG-1033-S) with an elution volume of 100 µL.

SARS-CoV-2 qRT-PCR was performed using the SARS-CoV-2 RT-qPCR kit (PerkinElmer; cat. no. 3501-0010). Using primers provided in the kit, SARS-CoV-2 Orf1ab and nucleocapsid (N) gene were amplified as targets according to the manufacturer’s instructions and along with nCoV internal standard, nCoV positive, nCoV negative controls provided in the kit.

## Results

### Aerosol generating system particle mass, size, and distribution

**Figure 3:**
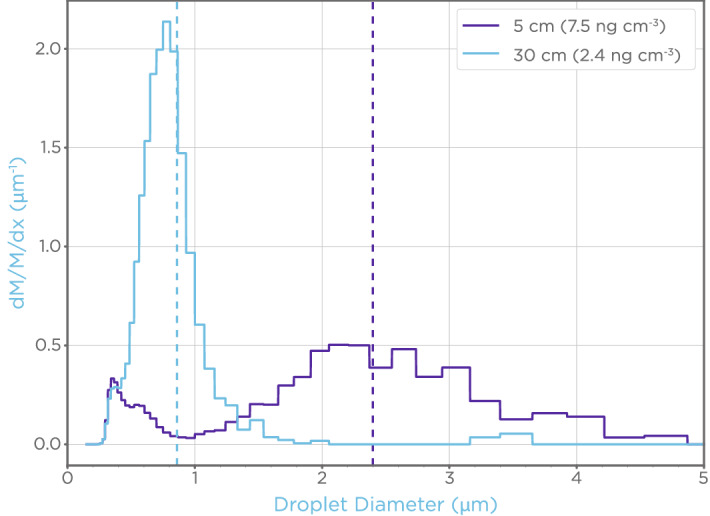
Particle mass distributions were measured based on two dilution path lengths. The particle probability density by mass (dM/M/dx) at a given particle diameter was measured by an optical particle counter placed 5 or 30 cm from the dilution region. The mass mean particle size (dashed line) was 2.4 µm and 0.9 µm when measured at 5 and 30 cm from the dilution region, respectively. The total collection efficiency by mass of the aerosol by the filter was > 99.8% (see Supplementary Fig. 4).

Prior to assessing the efficacy of virus capture by filters in the test rig, the aerosols produced by the OMROM NE-U780 ultrasonic nebulizer were characterized. This was accomplished using a Welas 2100 HP particle counter connected at two different path lengths, 5 cm and 30 cm, to measure the size of particles as influenced by evaporative loss over the distance traveled. Aerosols traversing the 5 cm path yielded a relatively broad distribution of sizes compared to the 30 cm path (Fig. 3). The respective aerosol concentrations of particles traversing the 5 cm and 30 cm paths were 7.5 and 2.4 ng/cm^3^, with mass mean diameters of 2.4 and 0.9 µm, respectively. Based on these results, it was calculated that 0.2 µL of aerosolized media from the nebulizer would be delivered over a period of 5 min, with evaporation differences across the two paths lengths changing the final particle size but not the number of virus particles delivered.

### Detection limit for aerosolized SARS-CoV-2 and HCoV-NL63

Six replicates of various serial dilutions of SARS-CoV-2 and HCoV-NL63 in DMEM were prepared and aerosolized onto filters for 5 min. Standard curves for qRT-PCR were generated and the resulting linear regression equations were used to calculate the number of copies of the respective viruses based on the measured Ct value. Relative to the expected number of virus copies, it was observed that the limits of detection for SARS-CoV-2 and HCoV-NL63 were each around 10 copies/filter (Table 1). DMEM blanks without virus did not yield a Ct value in qRT-PCR in either SARS-CoV-2 or HCoV-NL63 aerosolization studies.

**Table 1:** Quantitative RT-PCR results of aerosolized SARS-CoV-2 and HCoV-NL63 filter extract samples and calculated virus copies on filters.

Expected number of virus copies on filter	SARS-CoV-2		HCoV-NL63	
	Mean Ct values ± SD	Calculated number of copies on filter (mean ± SD) based on Ct value	Mean Ct values ± SD	Calculated number of copies on filter (mean ± SD) based on Ct value
1	Undetermined	Undetermined	Undetermined	Undetermined
10	39.9 ± 1.0^a^	16 ± 11	38.45 ± 1.3	14 ± 8
100	37.4 ± 1.53	127 ± 123	35.63 ± 1.3	100 ± 69
1000	33.4 ± 0.81	1437 ± 790	32.41 ± 0.4	801 ± 267

^a^For this dilution, the positive results were obtained in four samples out of six.

### Stability of SARS-CoV-2 on filters

**Figure 4:**
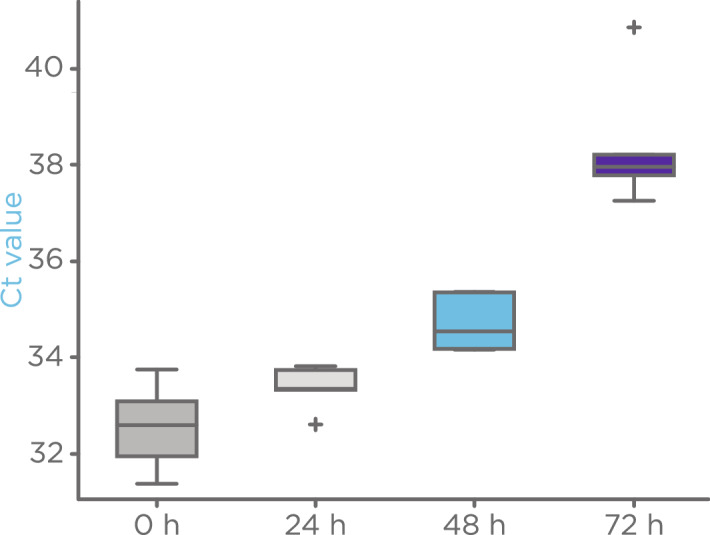
The stable half-life of inactivated SARS-CoV-2 on filters was close to 48 h. Virus samples aerosolized onto filters pre-exposed to breath for 30 min displayed an increase in Ct values from time 0 to 48 h that markedly increased between 48 and 72 h. There were 6 replicates per time point. Wilcoxon rank sum testing between time point 0 and time 24 h, 48 h and 72 h led to p-values of 0.25, 0.006 and 0.002 after correction for multiple testing, respectively. Filters not pre-exposed to breath showed similar stability (Supplementary Fig. 3).

The half-life of SARS-CoV-2 on filters, as evaluated by qRT-PCR, following aerosolization was studied to understand the stability of the virus on dry filters at ambient temperature. To investigate virus half-life, filter samples exposed to SARS-CoV-2 aerosols of 5000 copies/µL were immediately extracted with lysis buffer (0 h) or stored on filters at room temperature for 24 h, 48 h or 72 h before adding lysis buffer and storing at − 80 °C until qRT-PCR testing. The virus stability was tested in two different manners. The first experiment (n = 3) investigated the viral stability on dry filter and the second experiment (n = 6) additionally tested whether viral stability on filter was influenced by prior exposure of the filter to breath. Prior to virus aerosolization in the second experiment, face mask filters were exposed to exhaled breath aerosols for 30 min, dried, then used for aerosolized virus stability testing. As shown in Fig. 4 for breath pre-exposed filters, the Ct values for SARS-CoV-2 qRT-PCR showed an increasing trend from time 0 to 48 h. Translated into copy numbers, the Ct values at 0 and 48 h correspond to 1511 and 1008 on-filter copies of SARS-CoV-2, respectively, compared to the expected on-filter copy number of 1000. Although, the Wilcoxon rank sum test indicated statistical differences between time 0 and 48 h (p-value 0.006), the Ct values obtained at 48 h suggest that a sufficient viral load is present to enable positive detection of the SARS-CoV-2 on the filter. These results indicate that the stable half-life of SARS-CoV-2 on filters is close to 48 h at room temperature. Filters lacking pre-exposure to breath displayed stable Ct values up to 48 h which then markedly increased by 72 h (Supplementary Fig. 3).

### Detection of SARS-CoV-2 on face mask filters of COVID-19 patients

**Figure 5:**
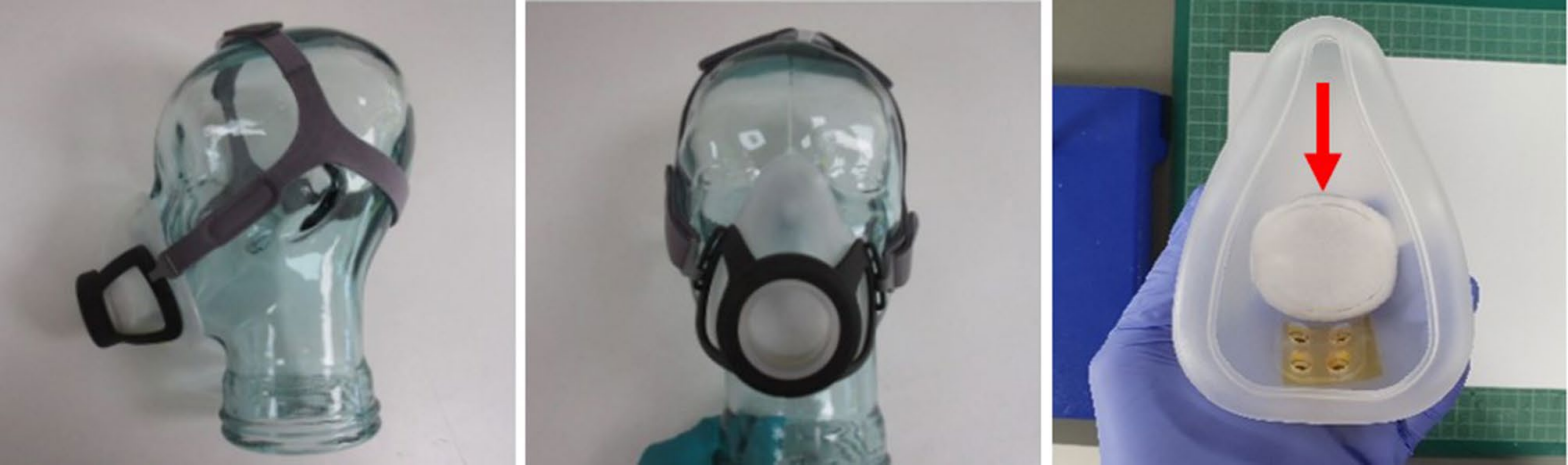
A filter held by a CPAP-type face mask was used to capture exhaled virus particles. The face mask, holder and head strap shown on a glass head in profile (left) and face on (center). An inside view of the face mask (right) with electrostatic filter (red arrow); holes at the bottom were sealed with tape.

Hospitalized patients with prior confirmed COVID-19 diagnosis were invited to participate in this study, and 48 subjects who provided consent were enrolled for the collection of breath samples. Out of those collected, samples from 1 patient were excluded from the final analysis due to the failure to recover any liquid volume from the electrostatic filter treated with DNA/RNA Shield, and follow-up NPS samples were not available for 8 subjects. Exhaled breath samples were collected by breathing through a face mask containing an embedded electrostatic filter (Fig. 5). The average age of the 47 patients included in the final analysis was 63 years, and 14 were female and 33 were male (Table 2). Forty percent of participants reported a cardiovascular comorbidity, more than a third had pulmonary comorbidities, and about one-fifth had diabetes. Shortness of breath (average duration 10.8 days), cough (average duration 11.5 days), and fever (average duration 6.9 days) were present in 81%, 57%, and 26% of patients. Computed tomography (CT) scans were performed on 29 of the 47 patients, with 90% having CT findings indicative of COVID-19 (Supplementary Data).

**Table 2:** Characteristics of COVID-19 patients.

	All included patients (n = 47)	Patients with a positive COVID-19 result from filter (TP, n = 4)	Patients with a negative COVID-19 result from filter (FN, n = 43)
Age in years, mean ± SD (median)	63 ± 15 (63)	38 ± 16 (38)	65 ± 13 (63)
Female/male	14/33	2/2	12/31
**Comorbidities and symptoms**			
Cardiovascular comorbidity (%)	19 (40%)	1 (25%)	18 (42%)
Pulmonary comorbidity (%)	16 (34%)	0 (0%)	16 (37%)
Diabetes (%)	10 (21%)	0 (0%)	10 (23%)
Shortness of Breath (n, days, %)	38, 10.8, 81%	3, 4.0, 75%	35, 11.3, 81%
Cough (n, days, %)	27, 11.5, 57%	1, 7, 25%	26, 11.7, 60%
Fever (n, days, %)	12, 6.9, 26%	3, 3.7, 75%	9, 8.0, 21%
**Other test results**			
COVID Positive CT Scan	26/29 (90%)	2/2 (100%)	24/27 (89%)

*TP* true positive, *FN* false negative.

Per protocol all patients underwent NPS and face mask sampling on the same day, except for 8 cases for which follow-up NPS samples were not collected. All patients (n = 47) in the trial were admitted to the hospital with COVID-19 based on a positive NPS PCR result for SARS-CoV-2. Many patients were diagnosed outside the hospital; the initial date of a positive NPS COVID-19 qPCR diagnosis was known for 20 of 47, and, for 8 of those 20, there was a lag of ≥ 5 days before a breath sample was collected on face mask filters with an average time of 5.0 ± 4.2 (mean ± SD) days between the initial NPS result and collection of samples on face mask filters (Supplementary Data).

Face mask filter sampling time averaged 50.3 ± 16.7 (mean ± SD) minutes (Supplementary Data); a collection period of 30–60 min was targeted but the collection could be discontinued by the patients at any time. RNA was isolated from filters and tested for SARS-CoV-2 by qRT-PCR, where 40 was the Ct cut-off for a SARS-CoV-2 qRT-PCR result to be considered positive. Four out of 47 (4/47) filter samples tested positive (Table 3) yielding a sensitivity of 8.5% with respect to the initial NPS SARS-CoV-2 qPCR test result. The patients with a positive SARS-CoV-2 test result from filter testing tended to be younger than those with a negative filter result, mean of 38 and 65 years old, respectively, and, on average, had more recent onset of symptoms such as fever, mean of 3.7 versus 8.0 days and shortness of breath, mean of 4.0 versus 11.3 days, respectively, among those with symptoms (Table 2). However, statistical comparisons between the filter qRT-PCR positive and negative groups were not performed to avoid false discovery due to the low number of positive results and unbalanced group sizes.

**Table 3:** Diagnostic performance of face mask filters compared to initial NPS for SARS-CoV-2 detection by qRT-PCR.

All filter samples compared to admission COVID-19 NPS results (n = 47)	Actual	
	COVID Pos	COVID Neg
**Predicted**		
COVID Pos	4	0
COVID Neg	43	0
Sensitivity	8.5%	

Since most patients in the study were diagnosed outside the hospital, follow-up NPS samples were collected shortly after face mask filter samples to offset possible skewing of test results due to a lag between filter and NPS sample collection. Thirty-nine patients had follow-up NPS collected at the time of face mask filter sample collection, including 2 of the 4 subjects with a positive SARS-CoV-2 qRT-PCR result. Of those 39 patients, 34 follow-up NPS samples tested positive for SARS-CoV-2 by qRT-PCR whereas 3 of the follow-up NPS samples were negative. Taking the follow-up NPS results as the standard of truth, there were 36 SARS-CoV-2 positive and 3 SARS-CoV-2 negative NPS qRT-PCR results compared to filter testing which yielded 2 true positives, no false positives, 34 false negatives, and 3 true negatives (Table 4) for a sensitivity of 5.6%.

**Table 4:** Diagnostic performance of face mask filters compared to follow-up NPS for SARS-CoV-2 detection by qRT-PCR. Filter samples compared to follow-up COVID-19 NPS result.

Filter samples compared to available follow-up COVID-19 NPS results (n = 39)	Actual	
	COVID Pos	COVID Neg
**Predicted**		
COVID Pos	2	0
COVID Neg	34	3
Sensitivity	5.6%	

The average qRT-PCR Ct value for the 36 SARS-CoV-2 positive follow-up NPS samples was 34.69 ± 4.84 (mean ± SD). There was a noticeable difference in Ct values between NPS and filters for the two patients with positive SARS-CoV-2 from filters; one patient had respective NPS and filter Ct values of 23.40 and 37.17 while the other had values of 28.37 and 39.95. Overall, the 4 filter samples with positive SARS-CoV-2 test results, irrespective of whether a matched follow-up NPS was available, had an average Ct of 35.60 ± 3.88 (mean ± SD).

## Discussion

In the present study, we investigated whether, in addition to preventing SARS-CoV-2 virus spread, filters embedded in some types of face masks and respirators could be used for SARS-CoV-2 detection. However, the sensitivity of SARS-CoV-2 detection when using samples extracted from filters was less than 10% of that of the nasopharyngeal swab samples used as a benchmark in this study.

Face mask filters are effective at capturing aerosols—even particles with submicron diameters (Supplementary Fig. 4)—and, using inactivated aerosolized SARS-CoV-2, we demonstrated a limit of detection on filters of around 10 copies/filter (Table 1). Furthermore, SARS-CoV-2 was detected on face mask filters for up to 48 h when stored dry at room temperature. Others have shown that SARS-CoV-2 can survive on dry filter paper at room temperature but with severely reduced infectivity by day 3; it is worth noting that qRT-PCR Ct values increased progressively from day 0 in that study^21^.

Previous work with exhaled breath captured on filters indicated that a small percentage (24%) of patients infected with human rhinovirus are high particle producers (HPP) whereas the majority are low particle producers (LPP)—with respective geometric means for the HPP and LPP groups of 3500 and 7.4 particles per liter of exhaled breath^15^. The authors mused that if the small airways of HPPs were infected with virus, they would be more likely to generate infectious aerosols and be likely super-spreaders. However, in their study, as in ours, no follow-up epidemiological contact tracing information was available to evaluate a possible HPP-superspreading link. Although virus particles were detected by the optical particle counter in their breath collection system and infection was confirmed by nasal lavage, no rhinovirus RNA was detected within the limits of the qPCR assay.

Similarly, an optimized PCR detection method identified influenza RNA in just 4 of 12 breath samples collected on Teflon filters from influenza patients, which translated to a detection range of < 48 to 300 copies of influenza virus RNA per filter in the 4 positive samples^16^. The authors showed that > 99% of the exhaled particles were < 5.0 µm in diameter which suggested that influenza virus may be contained in fine aerosols generated by tidal breathing. The low rate of detection of SARS-CoV-2 from COVID-19-confirmed patients in our study closely mirrors the detection rate of rhinovirus and influenza RNA on filters in these previous studies.

The patients with positive SARS-CoV-2 qRT-PCR results from filters were generally younger and had a more recent onset of fever and shortness of breath than the patients with negative qRT-PCR filter results. SARS-CoV-2 viral load peaks about 1 day before onset of symptoms and remains relatively high for first 3–4 days after symptom onset^22,23^. This suggests that respiratory droplets captured on filters from patients at the highest level of virus shed can be used for SARS-CoV-2 detection.

Operationally, several investigators have adopted indirect classifications of SARS-CoV-2 viral load based on NPS qPCR Ct values where Ct < 25, 25–30, or > 30 have been respectively defined as high, intermediate, or low viral load^24,25^ while others have expressed definitions as airborne concentrations^26^, or copies of viral RNA shed^27^. However, it must be stressed that Ct values reflect viral RNA levels and may not be a true indication of infectiousness^25^. Ct values are influenced by respiratory shedding rate and natural history of infection^25^, and, problematically, SARS-CoV-2 viral load peaks before symptoms appear^22,23^. However, a study of 100 PCR-confirmed COVID-19 patients in Singapore found that PCR Ct values ≤ 30 were significant predictors of viral culture isolation, duration of sickness, and indicated that lower PCR Ct values early in illness was associated with virus viability^28^. At least one report has shown a contradictory trend where non-hospitalized COVID-19 patients had higher viral loads than patients who were admitted to the hospital, but it was noted that the non-hospitalized patients were tested, on average, on day 3 of symptoms whereas the hospitalized cohort averaged 5 days^29^.

While the results show that SARS-CoV-2 testing of filters is less sensitive than NPS, there are limitations to the study worth noting. The electrostatic filters used in this study readily absorb liquid and, compared to NPS, required a fourfold greater volume to recover enough liquid to enable RNA extraction. Three patients had matched filter and NPS samples that yielded Ct values (Supplementary Data). In the case of the filters, two of the Ct values were below 40 and considered as positive SARS-CoV-2 results whereas one was above 40 and was defined as negative. The average Ct values for these pairs of filters and NPS were 39.85 ± 2.15 and 28.11 ± 3.75, respectively. In the absence of differences in extraction efficiency between filter and NPS samples, a fourfold dilution due to the different extraction volumes would account for 2 doubling cycles at most, but the difference observed between filters and NPS was 11.8 Ct units. These results indicate that the different volumes of DNA/RNA Shield used for storing samples were not the major factor contributing differences in sensitivity between filters and NPS. The number of days patients exhibited symptoms of fever, shortness of breath, and coughing averaged from 3.7 to 7 days in patients (n = 4) with positive SARS-CoV-2 qRT-PCR filter results but was 8.0–11.7 days in the filter negative group (n = 43) which indicates that the filter SARS-CoV-2 positive group was earlier in the infection cycle and at a higher point of virus emission^22,23^. Of the 4 COVID-19 patients with a positive SARS-CoV-2 filter result, 2 did not have a follow-up NPS sample for SARS-CoV-2 qRT-PCR testing and, overall, 8 of the enrolled patients did not have follow-up NPS samples available, so the final performance comparison matrix was based on fewer patients (Table 4). Although there was an apparent different in average age, 38 versus 66, respectively, between the SARS-CoV-2 qRT-PCR filter positive and negative groups, statistical testing between groups was not performed to avoid false discovery based on the small number of positive cases. Most patients were diagnosed with COVID-19 outside of the hospital in this study, and the time interval between the initial SARS-CoV-2 positive NPS sample and collection of the face mask filter sample was not known for 27 out of the 47 patients. Finally, a shortage of leakproof containers meant that 20 of the filters were frozen after collection rather than immersed in DNA/RNA Shield. While all 4 filters that tested positive for SARS-CoV-2 came from samples treated with DNA/RNA Shield following collection, a secondary research endpoint to examine the microbiome captured on face mask filters showed equivalent phyla representation on frozen versus DNA/RNA Shield-treated filters indicating similar nucleic acid integrity regardless of post-collection treatment.

During the writing of this manuscript, two important studies reported the use of face mask filters for detection of SARS-CoV-2^30,31^. In one study, 38% of polyvinyl-alcohol (PVA) strips embedded in face masks yielded positive SARS-CoV-2 qRT-PCR results in confirmed COVID-19 patients^30^ and 42% of dissolvable gelatin filters attached to N-95 face masks tested positive for SARS-CoV-2 by qRT-PCR in COVID-19 patients in the other study. While the rate of positive SARS-CoV-2 detection of filters was higher in both studies compared to the present work, it was noted that patients were predominantly sampled within 24^30^ to 36^31^ h from first known positive NPS SARS-CoV-2 result which differs from our study where a majority of patients were first diagnosed outside of the hospital. The number of days since onset of symptoms and viral load detected on PVA face mask filters were inversely correlated and the 4.2 day average onset of symptoms in COVID-19 with positive PVA SARS-CoV-2 qRT-PCR results^30^ closely mirrors the average days of symptom onset in the 4 COVID-19 patients with positive SARS-CoV-2 qRT-PCR results in our study (Table 2). As with our study, SARS-CoV-2 qRT-PCR Ct values were higher on filters compared to matched NPS from the same subject^31^. Altogether, these results underscore that the ability to detect SARS-CoV-2 on face mask filters is restricted to the early disease course when exhaled viral load is near a maximum.

Interestingly, while face mask filters do not offer enough sensitivity for practical SARS-CoV-2 (this study), rhinovirus^15^, and influenza^16^ detection, there is robust evidence for face mask sampling for the detection of *Mycobacterium tuberculosis.* A recent prospective observational study showed that face mask sampling on a gelatin filters was an efficient, non-invasive method for detecting *M. tuberculosis* and did so with greater consistency and at an earlier disease stage than sputum sampling^32^. Thus, there is potential for face mask filter sampling to diagnose tuberculosis and other bacterial infections and the ability to do so non-invasively holds promise for community-based testing.

## Conclusions

Spread of SARS-CoV-2 in respiratory droplets comprised of fine and coarse aerosols is an important route of transmission^22,23^. Although, the limit of detection of filters in this study was close to 10 copies/filter, diagnostic testing of face mask filters showed poor concordance between filters and NPS samples in the qRT-PCR detection of SARS-CoV-2 in confirmed COVID-19 patients. We detected SARS-CoV-2 RNA on 4 of 47 face mask filters worn by confirmed COVID-19 patients. The results indicate that face mask filters are not sensitive for routine SARS-CoV-2 detection in COVID-19 patients, but, as reported previously for rhinovirus^15^ and influenza^16^, SARS-CoV-2 can be detected in some instances. The lesser sensitivity indicates that face mask filters are inferior to NPS for detection of SARS-CoV-2. Future work may reveal greater diagnostic sensitivity of face mask filters for SARS-CoV-2 detection in pre-symptomatic populations or patients infected with bacteria or fungi such as *Mycobacterium tuberculosis*, *Aspergillus* species, or *Pseudomonas aeruginosa*.

## Supplementary Information

Supplementary Information 1.

Supplementary Information 2.

## Data Availability

All data generated or analyzed during this study are included in this published article (and its Supplementary Information and Supplementary Data files).
