# Supplementary material for: The SARS-CoV-2 viral load in COVID-19 patients is lower on face mask filters than on nasopharyngeal swabs
Source: Sci Rep. 2021 Jun 29;11:13476. doi: 10.1038/s41598-021-92665-3 (PMC8242000; doi:10.1038/s41598-021-92665-3)
Supplement: Supplementary file 1 — Supplementary Information 1. [file 41598_2021_92665_MOESM1_ESM.pdf]

Supplementary Information

The SARS-CoV-2 viral load in COVID-19 patients is lower on face mask filters than on nasopharyngeal swabs

Agnieszka Smolinska, David S. Jessop, Kirk L. Pappan, Alexandra De Saedeleer, Amerjit Kang, Alexandra L. Martin, Max Allsworth, Charlotte Tyson, Martine P. Bos, Matt Clancy, Mike Morel, Tony Cooke, Tom Dymond, Claire Harris, Jacqui Galloway, Paul Bresser, Nynke Dijkstra, Viresh Jagesar, Paul H.M. Savelkoul, Erik V.H. Beuken, Wesley H.V. Nix, Renaud Louis, Muriel Delvaux, Doriane Calmes, Benoit Ernst, Simona Pollini, Anna Peired, Julien Guiot, Sara Tomassetti, Andries E. Budding, Frank McCaughan, Stefan J. Marciniak, Marc P. van der Schee

Supplementary Information

Aerosol generating system in the laboratory

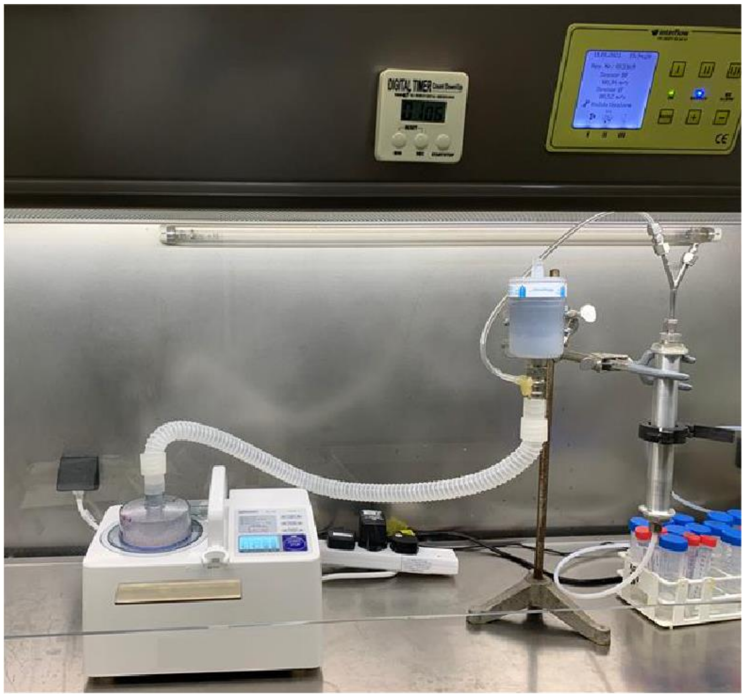

Supplementary Figure 1. Laboratory set up of the particle aerosolization system used for dispersing virus onto filters.

Effect of immediate RNA extraction from filters or after frozen storage

To investigate whether a limited duration freezing and thawing of filters prior viral RNA extraction had significant effect on the outcome, 500 copies/µL of inactivated SARS-CoV-2 were aerosolized and captured on filters (n=10). After aerosolization, the filters were divided into two groups. One group of filters (n=5) was immediately treated with lysis buffer for viral RNA extraction, while the remaining five filters were stored frozen for 5 days before extracting viral copies from the filters.

Quantitative-RT-PCR testing of filters stored in a dry, frozen state did not yield statistically significance differences (p-value of 0.7) to filters extracted immediately following aerosolization of inactivated SARS-CoV-2 (Supplementary Figure 2).

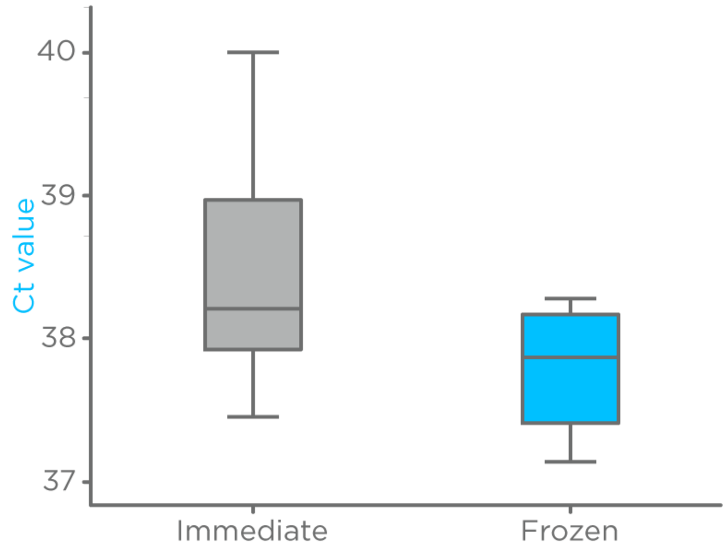

Supplementary Figure 2. Storage of filters in the dry, frozen state did not yield different results from filters extracted immediately following aerosolization of inactivated SARS-CoV-2.

Inactivated SARS-CoV-2 was stable at room temperature for up to 48h on dry filters that were not exposed to breath

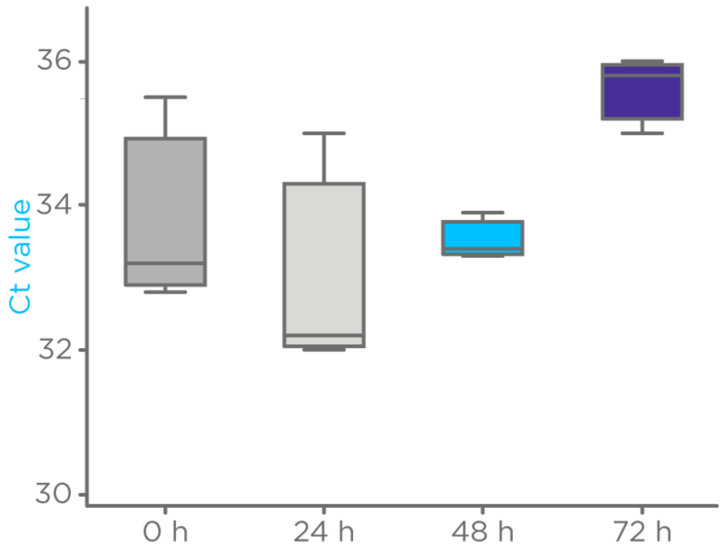

Supplementary Figure 3. SARS-CoV-2 was stable on filters for up to 48h. Filters not exposed to breath first showed stable Ct values for 48h which then increased at 72h. Wilcoxon rank sum testing between time point 0 and time 24h, 48h and 72h for filters not pre-exposed to breath (n=3) led to p-values to 0.3, 0.1 and 0.05, respectively. Results from filters that were first exposed to human breath before being spiked with inactivated SARS-CoV-2 are shown in Figure 4 in main manuscript.

Filter particle collection efficiency determination

The electrostatic filter collection efficiency was tested by measuring the aerosol particle distribution and concentration with and without the filter in place for 5 minutes at the full concentration (~$8x10^4$ particles cm^{-3}) of aerosol. The relative difference between the concentrations for each particle size bin is presented in Supplementary Figure 2. The minimum collection efficiency is > 98% and the total collection efficiency of the filter, for the particle size distributions in Figure 3 at 30 cm distance, is >99.8%.

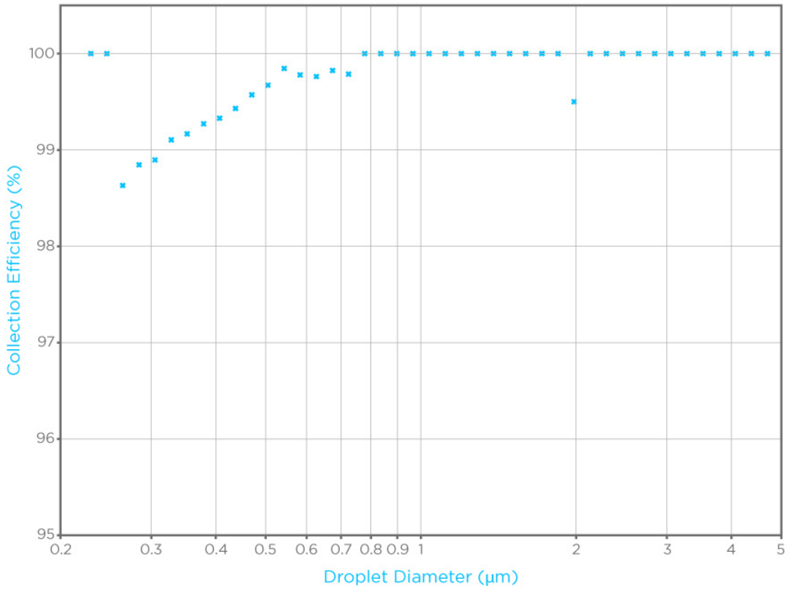

Supplementary Figure 4. Particle collection efficiency of the filter material for particles between 0.2 and 5.0 µm. Collection efficiency was 100% for all bins greater than 5.0 µm (measured up to 10.0 µm) but results were omitted from the graph.

Propagation and inactivation of coronaviruses

Supplementary Method 1. In vitro virus propagation and inactivation.

The SARS-CoV-2 and HCoV-NL63 viruses were cultured using monkey kidney cells (LLC-MK2) using Dulbecco's modified Eagle's Minimum Essential Medium (DMEM) 2% fetal bovine serum with gentamycin and mycostatin as culture medium. At time of harvesting 75% of cells were infected with the virus. After harvesting the virus was inactivated by heating for 2 hours at 60°C. To ensure the complete inactivation, the heat-treated viruses were re-cultured in LLC-MK2 cells. The cultured cell lines showed no cytopathogenic effects and the process was repeated in three independent batches to certify the inactivation of SARS-CoV-2.

Patient demographic data and test results

Supplementary Data. Patient data (.xlsx) and results for, "The SARS-CoV-2 viral load in COVID-19 patients is lower on face mask filters than on nasopharyngeal swabs."

NA, not applicable. nd, not done.
